# Heterotrophic Bacteria Enhance the Aggregation of the Marine Picocyanobacteria *Prochlorococcus* and *Synechococcus*

**DOI:** 10.3389/fmicb.2019.01864

**Published:** 2019-08-13

**Authors:** Bianca N. Cruz, Susanne Neuer

**Affiliations:** ^1^School of Life Sciences, Arizona State University, Tempe, AZ, United States; ^2^Center for Fundamental and Applied Microbiomics, Biodesign Institute, Arizona State University, Tempe, AZ, United States

**Keywords:** *Synechococcus*, *Prochlorococcus*, aggregation, bacteria, transparent exopolymeric particles

## Abstract

Marine picocyanobacteria are ubiquitous primary producers across the world’s oceans, and play a key role in the global carbon cycle. Recent evidence stemming from *in situ* investigations have shown that picocyanobacteria are able to sink out of the euphotic zone to depth, which has traditionally been associated with larger, mineral ballasted cells. The mechanisms behind the sinking of picocyanobacteria remain a point of contention, given that they are too small to sink on their own. To gain a mechanistic understanding of the potential role of picocyanobacteria in carbon export, we tested their ability to form “suspended” (5–60 μm) and “visible” (ca. > 0.1 mm) aggregates, as well as their production of transparent exopolymer particles (TEP)—which are a key component in the formation of marine aggregates. Additionally, we investigated if interactions with heterotrophic bacteria play a role in TEP production and aggregation in *Prochlorococcus* and *Synechococcus* by comparing xenic and axenic cultures. We observed TEP production and aggregation in batch cultures of axenic *Synechococcus*, but not in axenic *Prochlorococcus*. Heterotrophic bacteria enhanced TEP production as well as suspended and visible aggregate formation in *Prochlorococcus*, while in *Synechococcus*, aggregation was enhanced with no changes in TEP. Aggregation experiments using a natural plankton community dominated by picocyanobacteria resulted in aggregation only in the presence of the ballasting mineral kaolinite, and only when *Synechococcus* were in their highest seasonal abundance. Our results point to a different export potential between the two picocyanobacteria, which may be mediated by interactions with heterotrophic bacteria and presence of ballasting minerals. Further studies are needed to clarify the mechanistic role of bacteria in TEP production and aggregation of these picocyanobacteria.

## Introduction

The export of particulate organic carbon (POC) to ocean depth, a primary component of the marine carbon cycle, occurs in the form of sinking particles composed of aggregates of phytoplankton, bacteria, detritus, and inorganic matter. Marine picocyanobacteria of the genera *Prochlorococcus* and *Synechococcus* are the most abundant primary producers on Earth (Scanlan et al., 2009) and contribute up to 60% of the total phytoplankton carbon in oligotrophic open ocean regions (Durand et al., 2001). While they often co-occur, they have adapted to different ecological and biogeochemical conditions. *Synechococcus* are widespread in all marine environments from high latitudes to the tropics, and are more abundant in nutrient-rich than oligotrophic environments (Partensky et al., 1999). *Prochlorococcus*, despite their narrower geographical distribution, are more abundant in oligotrophic regions compared to *Synechococcus* (Partensky et al., 1999; Flombaum et al., 2013). The contribution of *Prochlorococcus* and *Synechococcus* to POC export remains unsettled due to their slow sinking rates as a result of their small size and lack of natural ballasting minerals (Michaels and Silver, 1988). Research in the past decade have suggested the importance of picophytoplankton (<2 μm), including picocyanobacteria, in carbon export, especially in oligotrophic ocean regions (Richardson and Jackson, 2007; Brew et al., 2009; Lomas and Moran, 2011; Amacher et al., 2013; Guidi et al., 2016; De Martini et al., 2018). At the Bermuda Atlantic Time-Series Study (BATS) site, located in the oligotrophic Northwestern Sargasso Sea, *Synechococcus* was overrepresented in sinking particles compared to the water column, while the opposite was observed for *Prochlorococcus* (Amacher et al., 2013), suggesting a differential contribution from these two picocyanobacteria to POC flux. The differential contribution of these two picocyanobacteria to POC flux was quantitatively confirmed by De Martini et al. (2018).

Sinking aggregates develop within a matrix of sticky organic substances known as transparent exopolymer particles (TEP). TEP are a class of exopolymers rich in acidic polysaccharides that form biotically or abiotically by exudation or coagulation of exopolymeric precursors (exopolymeric substances, EPS) (Alldredge et al., 1993; Passow and Alldredge, 1994). TEP production has been reported in large (>5 μm) species of diatoms (Fukao et al., 2012; Chen and Thornton, 2015), coccolithophores (Claquin et al., 2008; Van Oostende et al., 2013), as well as in filamentous and colonial cyanobacteria (Berman-Frank et al., 2007; Sohm et al., 2011; Pannard et al., 2016). Additionally, recent studies report marine *Synechococcus* as significant sources of TEP in various ocean regions (Ortega-Retuerta et al., 2019; Zamanillo et al., 2019), and *Prochlorococcus* to produce TEP in xenic batch cultures (Iuculano et al., 2017). Indeed, TEP production and subsequent aggregation with surrounding cells and inorganic particles are mechanisms for small phytoplankton to increase their effective size, density, and enhance their potential to sink to ocean depth (Burd and Jackson, 2009). Therefore, physical aggregation with surrounding cells and inorganic ballasting minerals is hypothesized as a pathway for the export of picophytoplankton cells beyond the euphotic zone (Richardson and Jackson, 2007; Richardson, 2019). Axenic batch cultures of *Synechococcus* produce TEP (Deng et al., 2016) and form sinking aggregates with the addition of kaolinite clay (Deng et al., 2015). Lithogenic clays, such as kaolinite, that are sourced from continental weathering and supplied to the oceans by long-range aeolic transport (Tosca et al., 2010), are commonly used for testing the role of ballasting minerals in phytoplankton aggregation (Hamm, 2002; Verspagen et al., 2006; Deng et al., 2015). Whether aggregation is also observed in *Prochlorococcus*, however, is still in question. To explain observations suggesting a differential contribution of *Synechococcus* and *Prochlorococcus* to POC flux, controlled laboratory studies are needed to test the potential mechanisms behind the export of these ubiquitous picocyanobacteria.

Although phytoplankton are known to be the most significant source of TEP, bacteria can also release TEP and/or its EPS precursors (Stoderegger and Herndl, 1999; Cho et al., 2004; Radić et al., 2006; Ortega-Retuerta et al., 2010, 2019). Furthermore, in contrast to EPS produced by phytoplankton (Hoagland et al., 1993; Bhaskar and Bhosle, 2005), the EPS produced by bacteria is higher in uronic acids, which make the surrounding environment, such as neighboring cells, more reactive with other surfaces (i.e., stickier) (Majumdar et al., 1999; Bhaskar and Bhosle, 2005). Thus, interactions between phytoplankton and bacteria can play a significant role in the formation and characteristics of sinking phytoplankton aggregates, controlling the fate of fixed carbon in aquatic ecosystems (Azam et al., 1994; Simon et al., 2002). While the influence of heterotrophic bacteria in diatom and coccolithophorid TEP exudation and aggregation has been studied (Grossart et al., 2006a,b; Gärdes et al., 2011; 2012; Van Oostende et al., 2013), their role in TEP production and aggregation in picophytoplankton, such as the globally-abundant picocyanobacteria, remains unknown.

We hypothesized that heterotrophic bacteria enhance the aggregation of marine *Prochlorococcus* and *Synechococcus* by influencing the production of TEP. We also hypothesized that *Prochlorococcus* have a lesser potential to form sinking aggregates compared to *Synechococcus*, even with the addition of ballasting minerals. To test these hypotheses, we monitored TEP production and aggregation throughout the growth of xenic and axenic cultures of *Prochlorococcus* and *Synechococcus*, and also tested their potential to form aggregates in a natural plankton community with and without the addition of ballasting minerals. Our study contributes to the understanding of the potential mechanisms of export of *Prochlorococcus* and *Synechococcus* to the deep ocean.

## Materials and Methods

### TEP and Aggregation in Batch Cultures

#### Growth of the Cyanobacteria

We grew duplicate 1 L batch cultures of marine *Synechococcus* sp. strains CCMP837 (WH7805, xenic), and CCMP2370 (WH8102, axenic) as well as *Prochlorococcus marinus* MED4 strains CCMP1986 (xenic), and CCMP2389 (axenic) obtained from the National Center for Marine Algae and Microbiota (NCMA) in 2 L Pyrex Erlenmeyer flasks. While not belonging to the same clade, the two *Synechococcus* cultures used in our study were both isolated from the Sargasso Sea and belong to the same phylogenetic cluster (cluster 5.1; Ahlgren and Rocap, 2012). Cultures were incubated on rocking platforms in a reach-in environmental growth chamber (Conviron) at 24 ± 1°C with a light intensity of 65–75 μmol photons m^–2^ s^–1^ in a 14 h:10 h light-dark cycle. *Synechococcus* and *Prochlorococcus* cells were grown in IMR medium (Eppley et al., 1967) and Pro99 medium (Moore et al., 2007), respectively, in artificial seawater (salinity 35, Kester et al., 1967). 50 mL samples were taken every other day until days 17–19, and immediately used to determine the volume of aggregates in the cultures as described below. Samples were then preserved in glutaraldehyde (1% (v/v) final concentration, Sigma-Aldrich) for TEP measurements, as well as the quantification of single cell abundance in cultures. All axenic cultures were tested for bacterial and fungal contamination at each sampling period by inoculation in IMR or Pro99 medium with added peptone and methylamine-HCl, as suggested by the NCMA. Axenic cultures were maintained as such throughout the experiments.

#### TEP Measurements

Transparent exopolymer particles concentrations in xenic and axenic picocyanobacteria cultures were determined as described in Passow and Alldredge (1995). 10 mL of glutaraldehyde-fixed culture samples (50 mL for Sargasso Seawater samples) were filtered through duplicate 0.4 μm pore-size polycarbonate membranes (GVS Life Technologies, ME, United States) at low and constant vacuum pressure (100 mm Hg). The retained TEP was subsequently stained with 0.5 mL of the acidic polysaccharide-specific Alcian Blue (AB) dye (8GX, Sigma-Aldrich), followed by a 0.5 mL rinse with MilliQ water for the removal of excess stain, and stored at −40°C until analysis. Prior to staining, the pre-calibrated 0.02% (w/v) AB working solution (provided by the Passow Lab, UCSB) with 0.06% (v/v) acetic acid (final pH 2.5) was passed through a 0.2 μm Acrodisc syringe filter (Pall Corporation, NY, United States) to remove undissolved dye. Membranes were soaked in 6 mL of 80% (v/v) sulfuric acid for 3 h to extract the AB-stained TEP and absorption was then measured using a spectrophotometer (Shimadzu UV-1601) at 787 nm. Duplicate stained filters with sterile media functioned as blanks. Although an improved AB calibration method has been reported (Bittar et al., 2018), the differences in calibration efficiency between the AB used in this study and that used by Bittar et al. (2018) are not significantly different based on tests performed by the Passow Lab (Uta Passow, personal communication). TEP concentrations were calculated using a calibration factor of the AB dye determined with xanthan gum (f-factor: 415) and expressed in μg of xanthan gum equivalent units per milliliter (μg XG eq. mL^–1^). TEP production and concentration in cultures were determined during their exponential phase of growth, thought to be representative of *in situ* dynamics, where they are actively growing, being grazed and supplied with recycled nutrients (Worden and Binder, 2003). Rates were calculated as in Iuculano et al. (2017). Furthermore, to compare picocyanobacteria TEP concentrations with phytoplankton of other cell sizes, we normalized TEP concentrations by cell biovolumes calculated by assuming simple geometrical shapes as in Passow (2002).

#### Determination of Single Cell Abundance and Suspended Aggregate Quantification

Single cell abundance in the cultures was determined with the use of epifluorescence microscopy (Carl Zeiss AxioScope.A1). Glutaraldehyde-fixed samples were stained with DAPI (4′,6-diamidino-2-phenylindole, 0.03M, Sigma-Aldrich), and filtered onto black 0.2 μm pore-size polycarbonate membranes (GVS Life Technologies, ME, United States). *Synechococcus* cells were visualized by their orange phycoerythrin fluorescence under blue-light excitation (450–490 nm), while DAPI stained *Prochlorococcus* and heterotrophic bacteria cells in xenic cultures were distinguished by their different cell morphologies under UV excitation (380–400 nm), cocci for *Prochlorococcus* versus bacilli for heterotrophic bacteria. To verify the accuracy of the epifluorescence counts, we compared cell counts of a serial dilution (1/10–1/1 × 10^4^) of a xenic *Prochlorococcus* culture obtained by microscopy with those obtained by flow cytometry (FCM). Briefly, 90–100 μL of Syto-9 (Thermo-Fisher Scientific, MA, United States) stained samples were ran on an Influx Mariner flow cytometer (Becton Dickinson, NJ, United States) using a forward-scatter (FSC) trigger, and detected using a 488 nm, 200 mW laser and a 531/40 emission filter. *Prochlorococcus* counts were determined as the difference between heterotrophic bacteria and total cell counts, and each FCM sample concentration was then determined using the volume-analyzed method (sample tube weighed before and after analysis) and expressed in cell number per mL. The cell abundance stemming from the FCM analyses of the picocyanobacteria and heterotrophic bacteria were not significantly different from the cell concentrations calculated from epifluorescence microscopy counts (*t*-test, *p* < 0.05 for *Prochlorococcus* and bacteria) (data not shown).

Concentrations of cell aggregates “suspended” in cultures [i.e., non-sinking particles with an equivalent spherical diameter (ESD) of 5–60 μm] were determined every other day throughout the 17–19-day incubation periods using a Multisizer 3 Particle Counter (Beckman Coulter, CA, United States). Prior to fixation with glutaraldehyde, samples in duplicates were diluted to a 1–10% final particle concentration with Isoton II diluent (Beckman Coulter, CA, United States) and aggregates were measured and quantified with a 100 μm aperture tube. The volume concentration of aggregates was calculated in μm^3^ per mL.

### Aggregation in Roller Tanks

#### Roller Tank Incubations With Batch Cultures

To investigate the formation of visible aggregates by xenic and axenic *Synechococcus* and *Prochlorococcus*, batch cultures were incubated in roller tanks to simulate the natural collision of particles as they would occur *in situ* (Shanks and Edmondson, 1989; Deng et al., 2015). 500 mL batch cultures were grown in the same conditions as the TEP experiments and incubated until the late exponential phase of growth was achieved (ca. 9 days). Cultures were then diluted to cell abundances simulating bloom conditions (10^6^ cells mL^–1^) and incubated in cylindrical 1.25 L Plexiglass roller tanks (Shanks and Edmondson, 1989) with artificial seawater (35‰, Kester et al., 1967). In addition to a control treatment with cells only, autoclaved kaolinite clay (Sigma-Aldrich) was dissolved in sterile artificial seawater and added to the cell suspensions in the roller tanks to final concentrations of 0.5 mg L^–1^ and 5 mg L^–1^. In each experiment, three treatments each with duplicate tanks were rotated on a rolling platform at 3.5 rotations per minute in the dark at 24°C for 7 days.

#### Roller Tank Incubations With a Natural Plankton Community

Forty-Five Liter of Sargasso Sea water was collected on the monthly BATS sampling cruises in fall 2017 (AE1718) and spring 2018 (AE1808) when *Prochlorococcus* and *Synechococcus* reached their peak abundances, respectively (Supplementary Table S1). Within 5 h of collection, the plankton community was incubated at the Bermuda Institute of Ocean Sciences, St. George’s, Bermuda, in walk-in chambers set to *in situ* temperature for 5 days, using the same experimental set-up as described above. The abundance of picocyanobacteria, picoeukaryotes, and heterotrophic bacteria were determined using FCM.

#### Determination of Aggregate Number, Size, Sinking Velocity, and Excess Density

Following the roller tank incubations, the number of visible aggregates (ca. > 0.1 mm) formed in each tank was counted and photographs of aggregates were taken with an Axiocam 105 color camera on a Discovery V20 stereo microscope for batch culture experiments, or on a Stemi 2000-C for field experiments (all by Carl Zeiss, Germany). The ESD of each imaged particle was then determined using ImageJ image analysis software^1^. To determine sinking velocities, aggregates from each roller tank were gently transferred with a wide-bore pipette into a 1 L settling cylinder and released at 1 cm under the air-water interface. The settling cylinder was filled with artificial seawater at the same salinity and temperature as roller tanks, and the settling time of each aggregate was determined through a vertical distance of 32.6 cm. Settling times were subsequently converted to velocities in meters per day. The excess density of aggregates was determined using the Navier-Stokes drag equation as in Iversen and Ploug (2010).

## Results

### TEP and Aggregation in Batch Cultures

#### Cell Abundance and Growth Dynamic*s*

**FIGURE 1:**
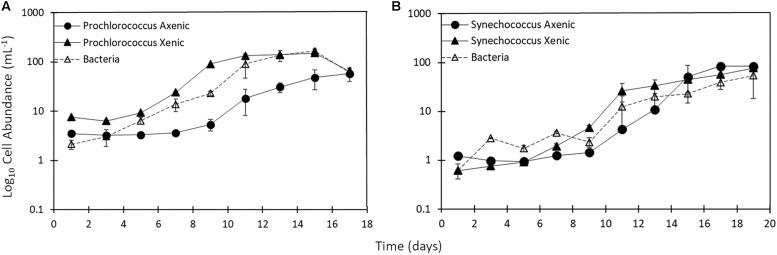
**(A)** Single cell abundance of *Prochlorococcus* (axenic – black circles; xenic – black triangles) as well as **(B)** *Synechococcus* (axenic – black circles; xenic – black triangles) and bacteria in corresponding xenic cultures (white triangles) throughout 17–19 day incubations. Error bars represent the standard error for duplicate cultures. Note error bars are smaller than the symbol sizes in some cases.

*Synechococcus* and *Prochlorococcus* cultures were sampled until days 17–19, when most cultures had reached the late exponential phase (Figures 1A,B). Growth rates of the picocyanobacteria were around 0.5 d^–1^ (Table 1). The growth of heterotrophic bacteria closely followed the growth of their associated picocyanobacteria (Figures 1A,B). Heterotrophs grew at rates of 0.33 ± 0.1 d^–1^ (mean ± standard error of *n* = 2 cultures) in *Synechococcus* and at 0.35 ± 0.04 d^–1^ in *Prochlorococcus* cultures (Table 1). Direct attachment between heterotrophs and picocyanobacteria cells could be observed by epifluorescence microscopy of xenic cultures, but no heterotrophic bacteria were observed in the axenic cultures.

**TABLE 1:** Growth rate, TEP production, and aggregate volume concentration during the exponential growth phase in xenic and axenic batch cultures of *Prochlorococcus* and *Synechococcus*.

**Culture**	**Growth rate**		**TEP production**	**Aggregate volume concentration**
		
	**Time interval (d)**	**Value (d^–1^)**	**Value (d^–1^)**	**Value (×10^6^ μm^3^ mL^–1^)**
*Prochlorococcus*, axenic	9–15	0.53 ± 0.05	–	0.38 ± 0.06
*Prochlorococcus*, xenic	5–9	0.57 ± 0.02	0.16 ± 0.08^*^	3.8 ± 0.37^*^
*Synechococcus*, axenic	9–17	0.50 ± 0.008	0.39 ± 0.06	7.6 ± 0.41
*Synechococcus*, xenic	5–11	0.55 ± 0.08	0.22 ± 0.08	9 ± 0.33^*^

Values are means ± the standard error of duplicate cultures. –, No data due to values being similar to blanks. ^*^Significantly different to its axenic counterpart (*t*-test, *p* < 0.05).

#### TEP

**FIGURE 2:**
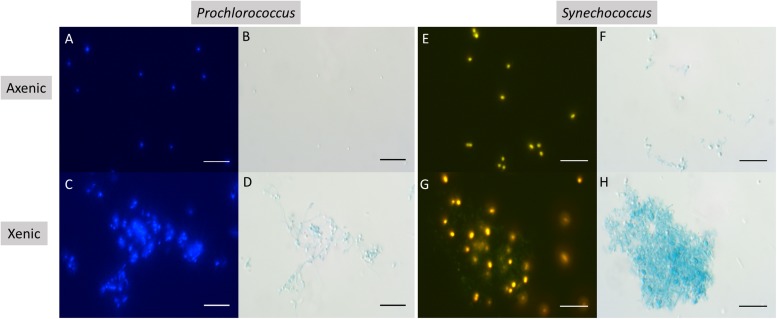
Epifluorescence **(A**,**C**,**E**,**G)** and corresponding brightfield **(B**,**D**,**F**,**H)** photomicrographs of Alcian Blue stained cultures of *Prochlorococcus* in axenic **(A**,**B)**, and xenic **(C**,**D)** conditions, as well as *Synechococcus* in axenic **(E**,**F)**, and xenic **(G**,**H)** conditions. Scale bars are 10 μm.

**FIGURE 3:**
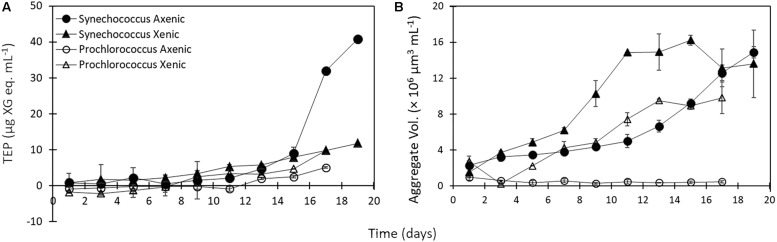
**(A)** TEP concentration throughout 17–19 day incubations of *Synechococcus* (axenic – black circles; xenic – black triangles), and *Prochlorococcus* (axenic – white circles; xenic – white triangles). **(B)** Volume concentration of aggregates present in the culture medium throughout 17–19 day incubations of *Synechococcus* (axenic – black circles; xenic – black triangles), and *Prochlorococcus* (axenic – white circles; xenic – white triangles). Error bars represent the standard error of duplicate cultures. Note error bars are smaller than the symbol sizes in some cases.

It was visually apparent that xenic *Prochlorococcus* as well as xenic and axenic *Synechococcus* cultures produced Alcian Blue-stainable exopolymeric material, i.e., TEP (Figure 2). TEP were accumulated in the cultures as the abundance of cells increased (Figures 1A,B, 3A). In axenic *Prochlorococcus*, TEP concentrations were low throughout the incubation period (0.71 ± 0.06 μg XG eq. mL^–1^, averaged across the 17-day experiment), with no production even during days of exponential growth (Table 1). In the xenic *Prochlorococcus* cultures, however, TEP concentration and production were measurable and significantly higher than in axenic cultures (*t-*test, *p* = 0.03 for TEP concentration, and *p* = 0.04 for TEP production; Tables 1, 2). In contrast, TEP concentration and production in xenic and axenic *Synechococcus* cultures did not differ significantly (*t-*test, *p* = 0.2 for both; Tables 1, 2). TEP production did not significantly differ between xenic *Prochlorococcus* and *Synechococcus* (*t*-test, *p* = 0.67; Table 1). TEP concentrations averaged across the exponential phase were two orders of magnitude higher in xenic *Synechococcus* than in *Prochlorococcus* (*t-*test, *p* = 0.01; Table 2).

**TABLE 2:** Comparison of cell volume-normalized TEP concentrations calculated in this study with xenic and axenic phytoplankton from the literature.

**Species**	**Cell volume-normalized TEP concentration (**×**10^–9^ μg XG eq. μm^–3^)**		**Study**
		
	**Xenic**	**Axenic**	
*Crocosphaera watsonii* (WH8501)	Up to 17	–	Sohm et al., 2011
*Emiliania huxleyi* (RCC1266)	295^*a*^	188^*a*^	Van Oostende et al., 2013
*Prochlorococcus marinus* (SS120)	2,682^*b*^	–	Iuculano et al., 2017
*Synechococcus* sp.	1,758 ± 278^*c*^	1,028 ± 337^*c*^	This study
*Prochlorococcus marinus* (MED4)	28 ± 3^*d*^	<1^*d*^	This study

*All data are from batch cultures in exponential growth phase. Values from this study are means ± the standard error of duplicate cultures. –, No data. Cell volumes were calculated assuming the following cell diameters: ^*a*^5 μm (Roscoff Culture Collection), ^*b*^0.6 μm (Roscoff Culture Collection), ^*c*^1 μm (NCMA), ^*d*^0.8 μm (NCMA).*

#### Suspended Aggregates

The presence of heterotrophic bacteria led to a higher volume concentration of suspended (5–60 μm) aggregates in *Synechococcus* and *Prochlorococcus* compared to axenic cultures (Figure 3B and Table 1). Additionally, the volume concentration of suspended aggregates correlated strongly with TEP concentrations, except in axenic *Prochlorococcus* (Supplementary Table S2). *Synechococcus* formed suspended aggregates in both xenic and axenic conditions, and the difference between the two cultures was significant throughout the experiment (6.5 ± 0.06 × 10^6^ μm^3^ mL^–1^ axenic and 9.9 ± 0.7 × 10^6^ μm^3^ mL^–1^ xenic, *t*-test, *p* = 0.03; Figure 3B). Conversely, the *Prochlorococcus* cultures did not aggregate throughout the experiment in axenic conditions, however, aggregates formed in the xenic cultures, especially during the exponential phase. The aggregate volume concentration in xenic cultures of *Synechococcus* and *Prochlorococcus* differed significantly throughout the 19-day incubation (*t*-test, *p* = 0.006; Figure 3B).

### Aggregation in Roller Tanks

#### Aggregation Using Picocyanobacteria From Batch Cultures

In addition to quantifying the development of suspended aggregates in batch cultures, we performed roller tank experiments to enhance the formation of visible, sinking aggregates. The picocyanobacteria cultures did not form visible aggregates unless 5 mg L^–1^ of kaolinite clay was added, except for axenic *Prochlorococcus*, which did not aggregate in any of the experimental treatments (Table 3). The number of aggregates formed by xenic and axenic *Synechococcus* cultures with 5 mg L^–1^ of kaolinite was not significantly different (*t*-test, *p* = 0.47; Table 3), but aggregates formed by the xenic cultures sank three times faster and were significantly larger (*t*-test, *p* < 0.001 for both; Table 3). Furthermore, xenic *Synechococcus* formed aggregates that sank faster and were significantly denser than those formed by xenic *Prochlorococcus* (*t*-test, *p* = 0.001, and *p* = 0.01, respectively; Table 3), although the number and size of aggregates formed did not significantly differ (*t*-test, *p* = 0.31, and *p* = 0.68, respectively; Table 3).

**TABLE 3:** Abundance, sinking velocity, size, and density of aggregates formed in roller tank experiments with increasing concentrations of kaolinite clay (control with no clay, treatments with 0.5, and 5 mg L^–1^).

**Culture**	**Background single cell abundance (× 10^6^ mL**^–^**^1^)**				**Aggregate abundance (L**^–^**^1^)**			**Sinking velocity (m d**^–^**^1^)**			**ESD (mm)**			**Excess density (×10**^–^**^3^ mg mL**^–^**^1^)**		
	
	**Before Rotation**	**Control**	**0.5 mg L^–1^**	**5.0 mg L^–1^**	**Control**	**0.5 mg L^–1^**	**5.0 mg L^–1^**	**Control**	**0.5 mg L^–1^**	**5.0 mg L^–1^**	**Control**	**0.5 mg L^–1^**	**5.0 mg L^–1^**	**Control**	**0.5 mg L^–1^**	**5.0 mg L^–1^**
*Prochlorococcus*, axenic	1.6 ± 0.4	1.4 ± 0.004	1.5 ± 0.05	1.0 ± 0.01	0	0	0	–	–	–	–	–	–	–	–	–
*Prochlorococcus*, xenic	*Pro*: 1.9 ± 0.2 Bacteria: 0.8 ± 0.03	*Pro*: 1.7 ± 0.02 Bacteria: 1.2 ± 0.1	*Pro*: 0.9 ± 0.04 Bacteria: 0.98 ± 0.04	*Pro*: 0.9 ± 0.02 Bacteria: 0.8 ± 0.06	0	0	2.4 ± 0.4^∗^	–	–	367 ± 137	–	–	3.8 ± 1.3	–	–	1.2 ± 0.5
*Synechococcus*, axenic	1.2 ± 0.1	1.0 ± 0.2	0.7 ± 0.03	0.7 ± 0.01	0	0	4.4 ± 3.6	–	–	743 ± 74	–	–	1.5 ± 0.3	–	–	10 ± 8
*Synechococcus*, xenic	*Syn*: 1.6 ± 0.08 Bacteria: 4.2 ± 0.6	*Syn*: 1.4 ± 0.1 Bacteria: 2.4 ± 0.4	*Syn*: 1.0 ± 0.15 Bacteria: 2.5 ± 0.3	*Syn*: 0.6 ± 0.03 Bacteria: 2.4 ± 0.1	0	0	1.2 ± 0.4	–	–	2273 ± 155^∗^	–	–	4.4 ± 0.6^∗^	–	–	15 ± 1.3

*Values are means ± the standard error of replicate tanks (aggregate abundance) or aggregates (all other parameters).–, No visible aggregates. ^∗^Significantly different to its axenic counterpart (*t*-test, *p* < 0.05).*

The number and sinking velocities of aggregates formed by axenic *Synechococcus* in our study differ from values in Deng et al. (2015). We looked into this discrepancy by testing the role of seawater media on the formation of aggregates in roller tanks using axenic *Synechococcus* (Supplementary Table S3) and found that the type of media influenced the number, ESD and sinking velocity of aggregates formed (One-way ANOVA, *p* < 0.05; Supplementary Table S3). No aggregates formed using artificial seawater (as used in our other experiments; ASW hereafter) or 0.2 μm-filtered natural seawater unless 5 mg L^–1^ of kaolinite clay was added. In contrast, aggregates formed in all roller tank treatments using artificial seawater made with Sigma Sea Salts as used by Deng et al. (2015), even without the addition of clay. Aggregates that formed with Sigma Sea Salts were an order of magnitude higher in abundance, sinking velocity, and excess density than those formed using ASW, however, these aggregates were also significantly smaller (*t*-test, *p* < 0.05 for all parameters; Supplementary Table S3). Roller tanks with 0.2 μm-filtered Sargasso Seawater formed fewer aggregates than did the other seawater treatments, but they were significantly larger, and sank at higher velocities compared to aggregates formed using ASW (*t*-test, *p* < 0.05 for all; Supplementary Table S3).

#### Aggregation Using a Natural Plankton Community

To test the aggregation of picocyanobacteria within a natural plankton community, we performed 5-day roller tank incubations of seawater collected at an open-ocean site off shore Bermuda, where picocyanobacteria are dominant primary producers (Supplementary Table S1). No visible aggregates formed at the end of incubations performed in September, when *Prochlorococcus* were in their highest abundance (8.6 ± 0.2 × 10^4^ cells mL^–1^), even with the addition of kaolinite clay. In March, when *Synechococcus* were in their highest abundance (1.8 ± 0.2 × 10^4^ cells mL^–1^), 5.6 ± 1.6 aggregates L^–1^ (*n* = 2 tanks) formed in tanks with 5 mg L^–1^ of kaolinite. The average ESD of the aggregates formed in March was 2.2 ± 0.55 mm (*n* = 6 aggregates) and aggregates sank at velocities of 2388 ± 17 m d^–1^ (*n* = 2 aggregates). Visualization of these aggregates using epifluorescence microscopy showed embedded phycoerythrin-rich cells (Supplementary Figure S1).

## Discussion

Our study provides, for the first time, a quantitative comparison of TEP production and aggregation in xenic and axenic *Prochlorococcus* and *Synechococcus*. In *Prochlorococcus*, we observed enhanced production of TEP and greater aggregation in xenic compared to axenic cultures, while *Synechococcus* cultures formed more aggregates in xenic conditions with no differences in TEP production, confirming our first hypothesis that heterotrophic bacteria enhance aggregation in these picocyanobacteria. Furthermore, compared to *Synechococcus*, *Prochlorococcus* formed fewer suspended (ESD 5–60 μm) aggregates, and visible (ca. > 0.1 mm) aggregates that formed in roller tanks sank significantly slower, despite the addition of ballasting minerals, suggesting a higher aggregation and export potential for *Synechococcus*. These results are consistent with roller tank experiments we conducted using seawater collected in the Sargasso Sea, where we only observed aggregation when *Synechococcus* were in their peak abundance (spring season, Supplementary Table S1), supporting our second hypothesis of *Synechococcus*’ higher propensity to form fast-sinking particles relative to *Prochlorococcus*.

The formation of visible aggregates in axenic *Synechococcus* only in the presence of added kaolinite contradicts findings reported by Deng et al. (2015), who found aggregation without the addition of kaolinite. These authors set up their roller tanks using artificial seawater made with Sigma Sea Salts, which in our tests of different seawater types (Supplementary Table S3) revealed significantly more and faster sinking aggregates compared to artificial seawater made as in Kester et al. (1967) used in this study, as well as when using just 0.2 μm-filtered natural seawater. It is likely that the enhanced aggregation reported by Deng et al. (2015), especially the aggregation they observed without the addition, and with lower concentration of kaolinite, was due to the addition of ballasting agents through precipitates formed as a byproduct of autoclaving the Sigma Sea Salts solution. These precipitates were likely carbonate minerals (Jones, 1967), which function as a ballast for aggregation as shown by Passow and De La Rocha (2006), similar to the kaolinite clay used in our study.

Aggregate formation is dependent on the number of particle collisions, and occurs as a function of the abundance of particles present in the medium (i.e., cell number) and their stickiness (i.e., the probability that cells stay attached after collision, for example due to the presence of TEP; Jackson, 1990; Burd and Jackson, 2009). Compared to axenic cultures, xenic cultures contain more particles due to the presence of heterotrophic bacteria, similar in size to the picocyanobacteria cells, thereby increasing the frequency of colliding particles in the medium. In addition, various studies using eukaryotic phytoplankton have shown that bacteria can also influence aggregation in xenic cultures by stimulating the production of TEP by phytoplankton (Grossart et al., 2006b; Gärdes et al., 2012; Van Oostende et al., 2013), and by increasing the stickiness of phytoplankton-derived EPS (Grossart et al., 2006b; Rochelle-Newall et al., 2010). In the case of the *Synechococcus* in our study, we know that the cells are inherently sticky because of the production of TEP (Figure 3 and Table 1) and formation of aggregates (Table 3) in axenic cultures. We postulate that either (1) heterotrophic bacteria present in xenic *Synechococcus* cultures increase the stickiness of *Synechococcus*-derived TEP or its precursors, and/or (2) the higher number of particles in xenic cultures leads to enhanced aggregation. In roller tank experiments using an order of magnitude higher cell number (ca. 10^7^ mL^–1^) of axenic *Synechococcus* cells, there was no enhanced aggregation observed compared to experiments performed using 10^6^ cells mL^–1^ (data not shown). This supports the first explanation above, that the bacteria’s influence on the aggregation is due to the modification of *Synechococcus*-derived TEP and not due to the increase in particle concentration.

In contrast to *Synechococcus*, the production of TEP in xenic *Prochlorococcus* cultures was more crucial in the formation of aggregates than particle abundance, as demonstrated by rapid aggregation once TEP was being produced (Figure 3, after day 7), and by the lack of visible aggregates in axenic *Prochlorococcus* despite the addition of kaolinite clay in roller tanks (Table 3). Hence, increasing particle abundance in axenic *Prochlorococcus* cultures did not result in enhanced aggregation, likely indicative of low cell stickiness due to low TEP. We hypothesize that the increased TEP and aggregation in xenic versus axenic *Prochlorococcus* is due to the heterotrophic bacteria exuding TEP and directly contributing to the TEP pool, or by stimulating its production in *Prochlorococcus*, thus enhancing aggregation due to increased particle stickiness.

Despite their small cell size, *Synechococcus* (xenic and axenic) and *Prochlorococcus* (xenic) cultures produced TEP in concentrations exceeding or comparable to other phytoplankton—such as the colonial marine cyanobacterium *Crocosphaera watsonii* (Sohm et al., 2011) and the coccolithophore *Emiliania huxleyi* (Van Oostende et al., 2013; Table 2). The enhanced TEP production in xenic versus axenic *Prochlorococcus* in our study suggests that the exudation of TEP by xenic *Prochlorococcus* observed by Iuculano et al. (2017) was likely due to the presence of heterotrophic bacteria rather than exudation solely by *Prochlorococcus*. The influence of heterotrophic bacteria on the aggregation and/or TEP production of picocyanobacteria, as seen here, complements findings on similar phytoplankton-bacteria interactions using diatoms and freshwater cyanobacteria. Grossart et al. (2006b) found that TEP and aggregation in batch cultures of the diatoms *Thalassiosira rotula* and *Skeletonema costatum* were differentially influenced by heterotrophic bacteria, enhancing aggregation in *T. rotula* but not in *S. costatum*. Interactions that enhance TEP production and aggregation have also been observed in the marine diatom *Thalassiosira weissflogii* in co-cultures with the bacterium *Marinobacter adhaerens* HP15, though the mechanism of this interaction has yet to be determined (Gärdes et al., 2011, 2012). Additionally, bacteria have been associated with increased EPS and TEP production in *Microcystis aeruginosa* (Pannard et al., 2016). Tight associations occur between marine heterotrophic bacteria and picocyanobacteria, such as the chemotactic attraction of bacteria to extracellular products of *Prochlorococcus* MED4 (Seymour et al., 2010), and the direct attachment of bacteria to *Synechococcus* cells (Malfatti and Azam, 2010; Zheng et al., 2018), demonstrating the potential for significant interactions between heterotrophic bacteria and picocyanobacteria. The specific roles of heterotrophic bacteria in the production of TEP and aggregation of xenic *Prochlorococcus* and *Synechococcus* needs to be elucidated in future studies by co-culturing each picocyanobacteria with representative heterotroph isolates.

While the above experiments, including our study, have been performed in nutrient balanced cultures (Redfield ratio, N:P = 16), it should be noted that TEP production could differ in the natural environment. TEP production by phytoplankton is enhanced in nutrient limited cultures (Corzo et al., 2000; Berman-Frank et al., 2007), and Deng et al. (2016) observed that axenic *Synechococcus* produced more TEP in nutrient limited (mainly nitrogen, N:P = 0.14) compared to nutrient balanced cultures. Additionally, the influence of bacteria on TEP production may also differ. Bacteria are found to stimulate TEP production in cultures under nutrient balanced conditions but not under nutrient limited conditions (Gärdes et al., 2012). Therefore, it is possible that the TEP dynamics observed in our study would differ under nutrient limitation, especially in oligotrophic open ocean settings. Nevertheless, our study points to the role of phytoplankton-bacteria interactions on TEP production and aggregate formation by *Synechococcus* and *Prochlorococcus* in the natural environment, where associations with bacteria would occur.

Despite higher cell abundances (10^6^ cells mL^–1^ and 10^4^ cells mL^–1^ for picocyanobacteria in culture-based and field-based experiments, respectively), the aggregation observed in roller tank experiments using picocyanobacteria cultures corroborate results from field experiments using Sargasso Seawater. In both cases, aggregates formed only when kaolinite clay was added (at a concentration of 5 mg L^–1^), and the number as well as sinking velocity of aggregates were within equal orders of magnitude. The observed increase in aggregate number and sinking velocity as a result of the addition of ballasting minerals is consistent with earlier aggregation studies with axenic *Synechococcus* (Deng et al., 2015), xenic diatoms (Passow and De La Rocha, 2006; De La Rocha et al., 2008), and natural plankton communities (Iversen and Robert, 2015; van der Jagt et al., 2018). Other aggregation studies on natural plankton communities in the North Sea (Iversen and Robert, 2015) and in the Cape Blanc upwelling (van der Jagt et al., 2018), dominated by diatoms, have observed aggregation in unballasted treatments and with a lower concentration of clay minerals (ca. 0.35 to 1 mg L^–1^). The silica frustules of the diatoms in those waters, as well as the high concentrations of lithogenic material off Cape Blanc, likely added mineral ballast that caused enhanced aggregation.

The higher sinking velocities of TEP-rich *Synechococcus*-derived aggregates, as well as the lack of aggregates formed when *Prochlorococcus* dominated the Sargasso Sea picocyanobacteria population, points to a higher export potential for *Synechococcus* versus *Prochlorococcus*. This has been hypothesized previously to explain the overrepresentation of *Synechococcus* in sequence libraries recovered from particle traps compared to water column libraries in the Sargasso Sea, in contrast to the *Prochlorococcus*, which were always underrepresented (Amacher et al., 2013; De Martini et al., in preparation). Furthermore, in a global regression-based modeling analysis of metagenomics data collected during the TARA Oceans project, Guidi et al. (2016) found *Synechococcus* to strongly correlate with carbon export in the subtropical oligotrophic ocean, but not *Prochlorococcus*. While De Martini et al. (2018) found that lower export of *Prochlorococcus* compared to *Synechococcus* was likely due to their smaller size, they also reported cases of higher absolute contribution of *Synechococcus* clades to sinking POC when compared to strains of *Prochlorococcus*. These authors also hypothesized that differences in aggregation, micro-grazer utilization and zooplankton mediation may play a role. Other mechanisms such as vertical mixing induced by internal solitary waves have been discussed in the literature to explain the observations of live picocyanobacterial cells in deep aphotic waters (Jiao et al., 2014), especially of *Prochlorococcus* (Jiao et al., 2014; Guo et al., 2018). But results of our study of enhanced stickiness and aggregate formation in *Synechococcus* due to their own TEP production, hint at a mechanism for their greater relative contribution to export in comparison to *Prochlorococcus*.

Small, suspended aggregates as formed in the picocyanobacteria cultures, would sink slowly *in situ* (following Stoke’s law) and therefore have a greater chance of being recycled in the euphotic zone (Lutz et al., 2002). On the other hand, small (11–64 μm) particles, the same size range as the suspended particles in our study (5–60 μm), were found to dominate particle flux at BATS (Durkin et al., 2015). Aggregation into suspended particles would increase *Prochlorococcus’* and *Synechococcus’* susceptibility to falling prey to mesozooplankton and may contribute to flux in their fast-sinking fecal pellets (Waite et al., 2000; Richardson and Jackson, 2007; Richardson, 2019). *Synechococcus* is an abundant constituent of zooplankton fecal pellets (Wilson and Steinberg, 2010; Stukel et al., 2013), however, this has not been observed for *Prochlorococcus*, which might be due to them forming fewer suspended aggregates that increase their functional size, and/or a more complete digestion because of their smaller size (Gorsky et al., 1999; Sutherland et al., 2010).

Our study is the first to show differences in TEP production and aggregation between xenic and axenic *Prochlorococcus* and *Synechococcus*. These results suggest a significant role for heterotrophic bacteria in TEP production and aggregation in *Prochlorococcus*, and also demonstrate that *Synechococcus* produce TEP and form aggregates independent of interactions with heterotrophic bacteria. Finally, we show for the first time that natural plankton communities from the Sargasso Sea do not form visible aggregates without the addition of ballast minerals, which confirms our experiments using picocyanobacteria cultures. Zooplankton utilization, possibly facilitated by the aggregation of the cyanobacteria, might be necessary to enable flux of these picocyanobacteria *in situ*.

## Data Availability

All datasets generated for this study are accessible through the Biological and Chemical Oceanography Data Management Office (https://www.bco-dmo.org/project/710239).

## Author Contributions

Both authors designed the study and wrote the manuscript. BC performed the experiments and analyzed the data.

## Conflict of Interest Statement

The authors declare that the research was conducted in the absence of any commercial or financial relationships that could be construed as a potential conflict of interest.

## Footnotes

1 http://rsb.info.nih.gov/ij/

## References

[B1] Ahlgren N. A. Rocap G. (2012). Diversity and distribution of marine *Synechococcus*: multiple gene phylogenies for consensus classification and development of qPCR assays for sensitive measurement of clades in the ocean. *Front. Microbiol.* 3:213. 10.3389/fmicb.2012.00213 22723796 PMC3377940

[B2] Alldredge A. L. Passow U. Logan B. E. (1993). The abundance and significance of a class of large, transparent organic particles in the ocean. *Deep. Res. Part I Oceanogr. Res. Pap.* 40 1131–1140. 10.1016/0967-0637(93)90129-q

[B3] Amacher J. Neuer S. Lomas M. (2013). DNA-based molecular fingerprinting of eukaryotic protists and cyanobacteria contributing to sinking particle flux at the Bermuda Atlantic Time-Series study. *Deep. Res. Part II Top. Stud. Oceanogr.* 93 71–83. 10.1016/j.dsr2.2013.01.001

[B4] Azam F. Smith D. C. Steward G. F. Hagström A. (1994). Sources of carbon for the microbial loop bacteria-organic matter coupling and its significance for oceanic carbon cycling. *Microb. Ecol.* 28 167–179. 10.1007/bf00166806 24186443

[B5] Berman-Frank I. Rosenberg G. Levitan O. Haramaty L. Mari X. (2007). Coupling between autocatalytic cell death and transparent exopolymeric particle production in the marine cyanobacterium *Trichodesmium*. *Environ. Microbiol.* 9 1415–1422. 10.1111/j.1462-2920.2007.01257.x 17504479

[B6] Bhaskar P. V. Bhosle N. B. (2005). Microbial extracellular polymeric substances in marine biogeochemical processes. *Curr. Sci.* 88 45–53.

[B7] Bittar T. B. Passow U. Hamaraty L. Bidle K. D. Harvey E. L. (2018). An updated method for the calibration of transparent exopolymer particle measurements. *Limnol. Oceanogr. Methods* 16 621–628. 10.1002/lom3.10268

[B8] Brew H. S. Moran S. B. Lomas M. W. Burd A. B. (2009). Plankton community composition, organic carbon and thorium-234 particle size distributions, and particle export in the Sargasso Sea. *J. Mar. Res.* 67 845–868. 10.1357/002224009792006124

[B9] Burd A. B. Jackson G. A. (2009). Particle aggregation. *Ann. Rev. Mar. Sci.* 1 65–90. 10.1146/annurev.marine.010908.163904 21141030

[B10] Chen J. Thornton D. C. O. (2015). Transparent exopolymer particle production and aggregation by a marine planktonic diatom (*Thalassiosira weissflogii*) at different growth rates. *J. Phycol.* 51 381–393. 10.1111/jpy.12285 26986532

[B11] Claquin P. Probert I. Lefebvre S. Véron B. (2008). Effects of temperature on photosynthetic parameters and TEP production in eight species of marine microalgae. *Aquat. Microb. Ecol.* 51 1–11. 10.3354/ame01187

[B12] Cho J.-C. Vergin K. L. Morris R. M. Giovannoni S. J. (2004). *Lentisphaera araneosa* gen. nov., sp. nov, a transparent exopolymer producing marine bacterium, and the description of a novel bacterial phylum, *Lentisphaerae*. *Environ. Microbiol.* 6 611–621. 10.1111/j.1462-2920.2004.00614.x 15142250

[B13] Corzo A. Morillo J. A. Rodríguez S. (2000). Production of transparent exopolymer particles (TEP) in cultures of *Chaetoceros calcitrans* under nitrogen limitation. *Aquat. Microb. Ecol.* 23 63–72. 10.3354/ame023063

[B14] De La Rocha C. L. Nowald N. Passow U. (2008). Interactions between diatom aggregates, minerals, particulate organic carbon, and dissolved organic matter: further implications for the ballast hypothesis. *Global Biogeochem. Cycles* 22 1–10. 10.1029/2007GB003156

[B15] De Martini F. Neuer S. Hamill D. Robidart J. Lomas M. W. (2018). Clade and strain specific contributions of *Synechococcus* and *Prochlorococcus* to carbon export in the Sargasso Sea. *Limnol. Oceanogr* 61 S448–S457. 10.1002/lno.10765

[B16] Deng W. Cruz B. N. Neuer S. (2016). Effects of nutrient limitation on cell growth, TEP production and aggregate formation of marine *Synechococcus*. *Aquat. Microb. Ecol.* 78 39–49. 10.3354/ame01803

[B17] Deng W. Monks L. Neuer S. (2015). Effects of clay minerals on the aggregation and subsequent settling of marine *Synechococcus*. *Limnol. Oceanogr.* 60 805–816. 10.1002/lno.10059

[B18] Durand M. D. Olson R. J. Chisholm S. W. (2001). Phytoplankton population dynamics at the Bermuda Atlantic Time-Series station in the Sargasso Sea. *Deep. Res. Part II Top. Stud. Oceanogr.* 48 1983–2003. 10.1016/S0967-0645(00)00166-1

[B19] Durkin C. A. Estapa M. L. Buesseler K. O. (2015). Observations of carbon export by small sinking particles in the upper mesopelagic. *Mar. Chem.* 175 72–81. 10.1016/j.marchem.2015.02.011

[B20] Eppley R. W. Holmes R. W. Strickland J. D. H. (1967). Sinking rates of marine phytoplankton measured with a fluorometer. *J. Exp. Mar. Bio. Ecol.* 1 191–208. 10.1016/0022-0981(67)90014-7 28972987

[B21] Flombaum P. Gallegos J. L. Gordillo R. A. Rincon J. Zabala L. L. Jiao N. (2013). Present and future global distributions of the marine cyanobacteria *Prochlorococcus* and *Synechococcus*. *Proc. Natl. Acad. Sci. U.S.A.* 110 9824–9829. 10.1073/pnas.1307701110 23703908 PMC3683724

[B22] Fukao T. Kimoto K. Kotani Y. (2012). Effect of temperature on cell growth and production of transparent exopolymer particles by the diatom *Coscinodiscus granii* isolated from marine mucilage. *J. Appl. Phycol.* 24 181–186. 10.1007/s10811-011-9666-3

[B23] Gärdes A. Iversen M. H. Grossart H.-P. Passow U. Ullrich M. S. (2011). Diatom-associated bacteria are required for aggregation of *Thalassiosira weissflogii*. *ISME J.* 5 436–45. 10.1038/ismej.2010.145 20827289 PMC3105730

[B24] Gärdes A. Ramaye Y. Grossart H. P. Passow U. Ullrich M. S. (2012). Effects of *Marinobacter adhaerens* HP15 on polymer exudation by *Thalassiosira weissflogii* at different N:P ratios. *Mar. Ecol. Prog. Ser.* 461 1–14. 10.3354/meps09894

[B25] Gorsky G. Chrétiennot-Dinet M. J. Blanchot J. Palazzoli I. (1999). Picoplankton and nanoplankton aggregation by appendicularians: fecal pellet contents of *Megalocercus huxleyi* in the equatorial Pacific. *J. Geophys. Res. Ocean.* 104 3381–3390. 10.1029/98jc01850

[B26] Grossart H. P. Czub G. Simon M. (2006a). Algae-bacteria interactions and their effects on aggregation and organic matter flux in the sea. *Environ. Microbiol.* 8 1074–1084. 10.1111/j.1462-2920.2006.00999.x 16689728

[B27] Grossart H. P. Kiorboe T. Tang K. W. Allgaier M. Yam E. M. Ploug H. (2006b). Interactions between marine snow and heterotrophic bacteria: aggregate formation and microbial dynamics. *Aquat. Microb. Ecol.* 42 19–26. 10.3354/ame042019

[B28] Guidi L. Chaffron S. Bittner L. Eveillard D. Larhlimi A. Roux S. (2016). Plankton networks driving carbon export in the oligotrophic ocean. *Nature.* 532 465–470. 10.1038/nature16942 26863193 PMC4851848

[B29] Guo R. Liang Y. Xin Y. Wang L. Mou S. Cao C. (2018). Insight into the pico- and nano-phytoplankton communities in the deepest biosphere, the Mariana Trench. *Front. Microbiol.* 9:2289. 10.3389/fmicb.2018.02289 30319587 PMC6168665

[B30] Hamm C. E. (2002). Interactive aggregation and sedimentation of diatoms and clay-sized lithogenic material. *Limnol. Oceanogr.* 47 1790–1795. 10.4319/lo.2002.47.6.1790

[B31] Hoagland K. D. Rosowski J. R. Gretz M. R. Roemer S. C. (1993). Diatom extracellular polymeric substances: function, fine structure, chemistry, and physiology. *J. Phycol.* 29 537–566. 10.1111/j.0022-3646.1993.00537.x

[B32] Iuculano F. Mazuecos I. P. Reche I. Agustí S. (2017). *Prochlorococcus* as a possible source for transparent exopolymer particles (TEP). *Front. Microbiol.* 8:709. 10.3389/fmicb.2017.00709 28491056 PMC5405065

[B33] Iversen M. H. Ploug H. (2010). Ballast minerals and the sinking carbon flux in the ocean: Carbon-specific respiration rates and sinking velocity of marine snow aggregates. *Biogeosciences.* 7 2613–2624. 10.5194/bg-7-2613-2010

[B34] Iversen M. H. Robert M. L. (2015). Ballasting effects of smectite on aggregate formation and export from a natural plankton community. *Mar. Chem.* 175 18–27. 10.1016/j.marchem.2015.04.009

[B35] Jackson G. A. (1990). A model of the formation of marine algal flocs by physical coagulation processes. *Deep Sea Res. Part I Oceanogr. Res. Pap.* 37 1197–1211. 10.1016/0198-0149(90)90038-W

[B36] Jiao N. Luo T. Zhang R. Yan W. Lin Y. Johnson Z. I. (2014). Presence of *Prochlorococcus* in the aphotic waters of the western Pacific Ocean. *Biogeosciences.* 11 2391–2400. 10.5194/bg-11-2391-2014

[B37] Jones G. E. (1967). Precipitates from autoclaved seawater. *Limnol. Oceanogr.* 12 165–167. 10.4319/lo.1967.12.1.0165

[B38] Kester D. R. Duedall I. W. Connors D. N. Pytkowicz R. M. (1967). Preparation of artificial seawater. *Limnol. Oceanogr.* 12 176–179. 10.4319/lo.1967.12.1.0176

[B39] Lomas M. W. Moran S. B. (2011). Evidence for aggregation and export of cyanobacteria and nano-eukaryotes from the Sargasso Sea euphotic zone. *Biogeosciences.* 8 203–216. 10.5194/bg-8-203-2011

[B40] Lutz M. Dunbar R. Caldeira K. (2002). Regional variability in the vertical flux of particulate organic carbon in the ocean interior. *Global Biogeochem. Cycles* 16 11–11. 10.1029/2000gb001383

[B41] Majumdar I. D’Souza F. Bhosle N. B. (1999). Microbial exopolysaccharides: effect on corrosion and partial chemical characterization. *J. Indian Inst. Sci.* 79 539–550.

[B42] Malfatti F. Azam F. (2010). Atomic force microscopy reveals microscale networks and possible symbioses among pelagic marine bacteria. *Aquat. Microb. Ecol.* 58 1–14. 10.3354/ame01355

[B43] Michaels A. F. Silver M. W. (1988). Primary production, sinking fluxes and the microbial food web. *Deep Sea Res. Part I Oceanogr. Res. Pap.* 35 473–490. 10.1016/0198-0149(88)90126-4

[B44] Moore L. R. Coe A. Zinser E. R. Saito M. A. Sullivan M. B. Lindell D. (2007). Culturing the marine cyanobacterium *Prochlorococcus*. *Limnol. Oceanogr. Methods* 5 353–362. 10.4319/lom.2007.5.353

[B45] Ortega-Retuerta E. Duarte C. M. Reche I. (2010). Significance of bacterial activity for the distribution and dynamics of transparent exopolymer particles in the Mediterranean Sea. *Microb. Ecol.* 59 808–818. 10.1007/s00248-010-9640-7 20221594

[B46] Ortega-Retuerta E. Mazuecos I. P. Reche I. Gasol J. M. Álvarez-Salgado X. A. Álvarez M. (2019). Transparent Exopolymer Particle (TEP) distribution and in situ prokaryotic generation across the deep Mediterranean Sea and nearby North East Atlantic Ocean. *Prog. Oceanogr*. 173 180–191. 10.1016/j.pocean.2019.03.002

[B47] Pannard A. Pédrono J. Bormans M. Briand E. Claquin P. Lagadeuc Y. (2016). Production of exopolymers (EPS) by cyanobacteria: impact on the carbon-to-nutrient ratio of the particulate organic matter. *Aquat. Ecol.* 50 29–44. 10.1007/s10452-015-9550-3

[B48] Partensky F. Blanchot J. Vaulot D. (1999). Differential distribution and ecology of *Prochlorococcus* and *Synechococcus* in oceanic waters: a review. *Bull. Inst. océanogr. (Monaco).* 19 457–475.

[B49] Passow U. (2002). Production of transparent exopolymer particles (TEP) by phyto- and bacterioplankton. *Mar. Ecol. Prog. Ser.* 236 1–12. 10.3354/meps236001

[B50] Passow U. Alldredge A. L. (1994). Distribution, size and bacterial colonization of transparent exopolymer particles (TEP) in the ocean. *Mar. Ecol. Prog. Ser.* 113 185–198. 10.3354/meps113185

[B51] Passow U. Alldredge A. L. (1995). A dye-binding assay for the spectrophotometric measurement of transparent exopolymer particles (TEP). *Limnol. Oceanogr.* 40 1326–1335. 10.4319/lo.1995.40.7.1326

[B52] Passow U. De La Rocha C. L. (2006). Accumulation of mineral ballast on organic aggregates. *Global Biogeochem. Cycles* 20 1–7. 10.1029/2005GB002579

[B53] Radić T. Ivančić I. Fuks D. Radić J. (2006). Marine bacterioplankton production of polysaccharidic and proteinaceous particles under different nutrient regimes. *FEMS Microbiol. Ecol.* 58 333–342. 10.1111/j.1574-6941.2006.00176.x 17117978

[B54] Richardson T. L. (2019). Mechanisms and Pathways of Small-Phytoplankton Export from the Surface Ocean. *Ann. Rev. Mar. Sci.* 11 57–74. 10.1146/annurev-marine-121916-163627 29996063

[B55] Richardson T. L. Jackson G. A. (2007). Small phytoplankton and carbon export from the surface ocean. *Science.* 315 838–840. 10.1126/science.1133471 17289995

[B56] Rochelle-Newall E. J. Mari X. Pringault O. (2010). Sticking properties of transparent exopolymeric particles (TEP) during aging and biodegradation. *J. Plankton Res.* 32 1433–1442. 10.1093/plankt/fbq060

[B57] Scanlan D. J. Ostrowski M. Mazard S. Dufresne A. Garczarek L. Hess W. R. (2009). Ecological genomics of marine picocyanobacteria. *Microbiol. Mol. Biol. Rev.* 73 249–299. 10.1128/MMBR.00035-8 19487728 PMC2698417

[B58] Seymour J. R. Ahmed T. Durham W. M. Stocker R. (2010). Chemotactic response of marine bacteria to the extracellular products of *Synechococcus* and *Prochlorococcus*. *Aquat. Microb. Ecol.* 59 161–168. 10.3354/ame01400

[B59] Shanks A. L. Edmondson E. W. (1989). Laboratory-made artificial marine snow: a biological model of the real thing. *Mar. Biol.* 101 463–470. 10.1007/bf00541648

[B60] Simon M. Grossart H. P. Schweitzer B. Ploug H. (2002). Microbial ecology of organic aggregates in aquatic ecosystems. *Aquat. Microb. Ecol.* 28 175–211. 10.3354/ame028175

[B61] Sohm J. A. Edwards B. R. Wilson B. G. Webb E. A. (2011). Constitutive extracellular polysaccharide (EPS) production by specific isolates of *Crocosphaera watsonii*. *Front. Microbiol.* 2:229. 10.3389/fmicb.2011.00229 22110469 PMC3215947

[B62] Stoderegger K. E. Herndl G. J. (1999). Production of exopolymer particles by marine bacterioplankton under contrasting turbulence conditions. *Mar. Ecol. Prog. Ser.* 189 9–16. 10.3354/meps189009

[B63] Stukel M. R. Décima M. Selph K. E. Taniguchi D. A. A. Landry M. R. (2013). The role of *Synechococcus* in vertical flux in the Costa Rica upwelling dome. *Prog. Oceanogr.* 112–113 49–59. 10.1016/j.pocean.2013.04.003

[B64] Sutherland K. R. Madin L. P. Stocker R. (2010). Filtration of submicrometer particles by pelagic tunicates. *Proc. Natl. Acad. Sci. U.S.A.* 107 15129–15134. 10.1073/pnas.1003599107 20696887 PMC2930554

[B65] Tosca N. J. Johnston D. T. Mushegian A. Rothman D. H. Summons R. E. Knoll A. H. (2010). Clay mineralogy, organic carbon burial, and redox evolution in Proterozoic oceans. *Geochim. Cosmochim. Acta.* 74 1579–1592. 10.1016/j.gca.2009.12.001

[B66] van der Jagt H. Friese C. Stuut J. B. W. Fischer G. Iversen M. H. (2018). The ballasting effect of Saharan dust deposition on aggregate dynamics and carbon export: aggregation, settling, and scavenging potential of marine snow. *Limnol. Oceanogr.* 63 1386–1394. 10.1002/lno.10779

[B67] Van Oostende N. Moerdijk-Poortvliet T. C. W. Boschker H. T. S. Vyverman W. Sabbe K. (2013). Release of dissolved carbohydrates by *Emiliania huxleyi* and formation of transparent exopolymer particles depend on algal life cycle and bacterial activity. *Environ. Microbiol.* 15 1514–1531. 10.1111/j.1462-2920.2012.02873.x 22985062

[B68] Verspagen J. M. H. Visser P. M. Huisman J. (2006). Aggregation with clay causes sedimentation of the buoyant cyanobacteria *Microcystis* spp. *Aquat. Microb. Ecol.* 44 165–174. 10.3354/ame044165

[B69] Waite A. M. Safi K. A. Hall J. A. Nodder S. D. (2000). Mass sedimentation of picoplankton embedded in organic aggregates. *Limnol. Oceanogr.* 45 87–97. 10.4319/lo.2000.45.1.0087

[B70] Wilson S. E. Steinberg D. K. (2010). Autotrophic picoplankton in mesozooplankton guts: evidence of aggregate feeding in the mesopelagic zone and export of small phytoplankton. *Mar. Ecol. Prog. Ser.* 412 11–27. 10.3354/meps08648

[B71] Worden A. Z. Binder B. J. (2003). Application of dilution experiments for measuring growth and mortality rates among *Prochlorococcus* and *Synechococcus* populations in oligotrophic environments. *Aquat. Microb. Ecol.* 30 159–174. 10.3354/ame030159

[B72] Zamanillo M. Ortega-Retuerta E. Nunes S. Rodríguez-Ros P. Dall’osto M. Estrada M. (2019). Main drivers of transparent exopolymer particle distribution across the surface Atlantic Ocean. *Biogeosciences.* 16 733–749. 10.5194/bg-16-733-2019

[B73] Zheng Q. Wang Y. Xie R. Lang A. S. Liu Y. Lu J. (2018). Dynamics of heterotrophic bacterial assemblages within *Synechococcus* cultures. *Appl. Environ. Microbiol* 84 e1517–17. 10.1128/AEM.01517-7 29150500 PMC5772231

