# Supplementary material for: Heterotrophic Bacteria Enhance the Aggregation of the Marine Picocyanobacteria Prochlorococcus and Synechococcus
Source: Front Microbiol. 2019 Aug 13;10:1864. doi: 10.3389/fmicb.2019.01864 (PMC6700329; doi:10.3389/fmicb.2019.01864)
Supplement: Supplementary file 1 [file Table_1.docx]

| Date | | **Station (lat-long)** | **Salinity** | **Temperature (℃)** | **TEP (µg XG eq. L^-1^)** | ***Prochlorococcus* (×10^4^ cells mL**^-^**^1^)** | ***Synechococcus* (×10^4^ cells mL**^-^**^1^)** | **Bacteria (×10^4^ cells mL**^-^**^1^)** | **Pico- & Nanoeukaryotes (×10^4^ cells mL**^-^**^1^)** |
| --- | --- | --- | --- | --- | --- | --- | --- | --- | --- |
| **16 Sept 2017** | |  |  |  |  |  |  |  |  |
| Depth (m) | 10 | 32°18.16 N, 64°34.51 W | 36.5 | 28.3 | 22.7 ± 4.3 | 8.6 ± 0.1 | 0.7 ± 0.3 | 20 ± 2 | - |
|  | 130 |  | 36.5 | 20.1 | 35 ± 1.8 | - | - | - | - |
| **16 Mar 2018** | | 31°58.77 N, 64°22.80 W | 36.6 | 21.1 | 24.4 ± 7.6 | 3.8 ± 0.4 | 1.8 ± 0.2 | 38 ± 5 | 0.2 ± 0.06 |
| Depth (m) | 50 |  |  |  |  |  |  |  |  |

**Supplementary Table 1**. Station information (sampling depth, salinity, temperature), TEP, and cell concentrations of picocyanobacteria, bacteria, and pico- and nanoeukaryotes in ambient seawater collected in the Sargasso Sea for roller tank incubations (mean ± the standard error of duplicate samples).

- : No data.
