# Supplementary material for: Heterotrophic Bacteria Enhance the Aggregation of the Marine Picocyanobacteria Prochlorococcus and Synechococcus
Source: Front Microbiol. 2019 Aug 13;10:1864. doi: 10.3389/fmicb.2019.01864 (PMC6700329; doi:10.3389/fmicb.2019.01864)
Supplement: Supplementary file 2 [file Table_2.DOCX]

**Supplementary Table 2**. Results of simple linear regression analyses between the volume concentration of suspended aggregates and TEP concentration during the exponential growth phase of xenic and axenic cultures of *Prochlorococcus* and *Synechococcus*. *n* = number of observations. *R*^2^ = explained variance. *P* = level of significance.

| **Culture** | ***n*** | ***R*^2^** | ***P*** | **Intercept ± SE** | **Slope ± SE** |
| --- | --- | --- | --- | --- | --- |
| *Prochlorococcus,*  axenic | 4 | 0.11 | 0.67 | 0.37 ± 0.05 | 0.013 ± 0.03 |
| *Prochlorococcus,*  xenic | 3 | 0.71 | 0.03 | 3.6 ± 0.61 | 0.59 ± 0.1 |
| *Synechococcus,*  axenic | 5 | 0.88 | 0.02 | 5.11 ± 0.8 | 0.25 ± 0.05 |
| *Synechococcus,*  xenic | 4 | 0.99 | 0.004 | 0.77 ± 0.56 | 2.67 ± 0.16 |
