# Supplementary material for: Heterotrophic Bacteria Enhance the Aggregation of the Marine Picocyanobacteria Prochlorococcus and Synechococcus
Source: Front Microbiol. 2019 Aug 13;10:1864. doi: 10.3389/fmicb.2019.01864 (PMC6700329; doi:10.3389/fmicb.2019.01864)
Supplement: Supplementary file 3 [file Table_3.DOCX]

**Supplementary Table 3**. Characteristics of visible (>0.1 mm) aggregates formed by axenic *Synechococcus* (10^6^ cells mL^-1^) incubated in roller tanks using different types of seawater in control and kaolinite clay amended treatments (concentration in mg L^-1^`). Values represent the mean ± the standard error of replicate tanks (for abundance) or aggregates (all other parameters).

| Seawater | **Abundance**  **(# of aggregates L^-1^)** | | | **Sinking Velocity (m d^-1^)** | | | **ESD (mm)** | | | **Excess Density (×10^-3^ g mL^-1^)** | | | |
| --- | --- | --- | --- | --- | --- | --- | --- | --- | --- | --- | --- | --- | --- |
|  | Control | 0.5 mg L^-1^ | 5.0 mg L^-1^ | Control | 0.5 mg L^-1^ | 5.0 mg L^-1^ | Control | 0.5 mg L^-1^ | 5.0 mg L^-1^ | Control | 0.5 mg L^-1^ | 5.0 mg L^-1^ |  |
| Sigma Sea Salts | 177 ± 23 | 229 ± 11 | 648 ± 352 | 798 ± 49 | 1007 ± 45 | 1227 ± 42 | 0.89 ± 0.08 | 0.88 ± 0.06 | 0.67 ± 0.14 | 36 ± 3 | 49 ± 1.9 | 100 ± 2.7 |  |
| Artificial Seawater | 0 | 0 | 8.4 ± 6.8 | - | - | 926 ± 39 | - | - | 1.7 ± 0.3 | - | - | 12 ± 8.5 |  |
| 0.2 μm-filtered Sargasso Sea water | 0 | 0 | 1.2 ± 0.4 | - | - | 3051 ± 262 | - | - | 4.3 ± 0.9 | - | - | 21 ± 4 |  |

- : No visible aggregates.
