# Supplementary material for: Heterotrophic Bacteria Enhance the Aggregation of the Marine Picocyanobacteria Prochlorococcus and Synechococcus
Source: Front Microbiol. 2019 Aug 13;10:1864. doi: 10.3389/fmicb.2019.01864 (PMC6700329; doi:10.3389/fmicb.2019.01864)
Supplement: Supplementary file 4 [file Image_1.pdf]

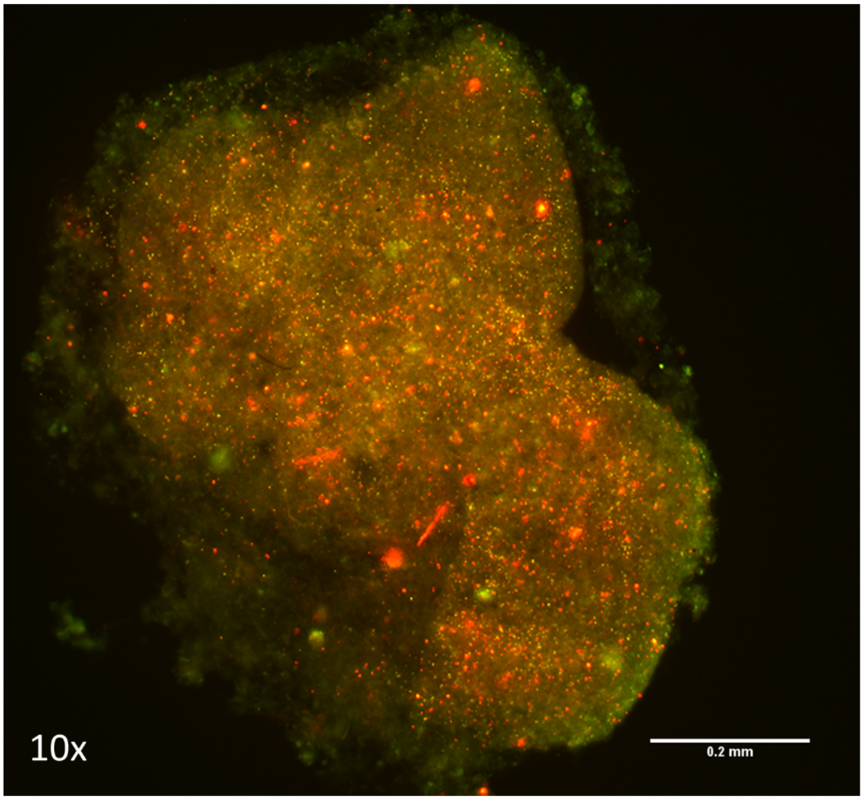

Supplementary Figure 1. Epifluorescence photomicrograph of an exemplary aggregate formed at the end of roller tank experiments using a natural plankton community from the Sargasso Sea with the addition of 5 mg L^{-1} kaolinite clay (refer to Table S1 for details on seawater sampling and collection). Imaged at 10x magnification. Red fluorescence: chlorophyll-a; yellow fluorescence: phycoerythrin (likely from *Synechococcus* sp.)
